# Training and Familiarization with Industrial Exoskeletons: A Review of Considerations, Protocols, and Approaches for Effective Implementation

**DOI:** 10.3390/biomimetics9090520

**Published:** 2024-08-30

**Authors:** Pranav Madhav Kuber, Ehsan Rashedi

**Affiliations:** Rochester Institute of Technology, 1 Lomb Memorial Dr, Rochester, NY 14623, USA; pmk2015@rit.edu

**Keywords:** wearable assistive devices, exoskeletons, motor learning, workforce training, human factors, instructional design, technology acceptance

## Abstract

Effective training programs are essential for safely integrating exoskeletons (EXOs) in industrial workplaces. Since the effects of wearable systems depend highly upon their proper use, lack of training of end-users may cause adverse effects on users. We reviewed articles that incorporated training and familiarization protocols to train novices on proper operation/use of EXOs. Findings showed variation in training methods that were implemented to train study participants in EXO evaluation studies. Studies also indicate that multiple (up to four) sessions may be needed for novice EXO wearers to match movement patterns of experts, and training can offer benefits in enhancing motor learning in novices. Biomechanical assessments and ergonomic evaluations can be helpful in developing EXO-specific training protocols by determining training parameters (duration/number of sessions and task difficulty). Future directions include development of personalized training approaches by assessing user behavior/performance through integration of emerging sensing technologies. Application of simulators and use of data-driven approaches for customizing training protocols to individuals, tasks, and EXO design are provided along with a comprehensive training framework. Discussed elements in this article can be helpful to exoskeleton researchers in familiarizing novice users to EXOs prior to evaluation, and to practitioners in developing protocols for training workforce.

## 1. Introduction

**Figure 1:**
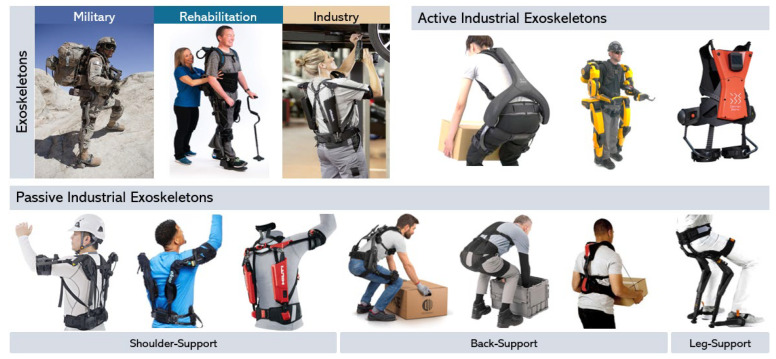
Application areas of exoskeletons (**top**-**left**), active (**top**-**right**) and passive (**bottom**) industrial exoskeletons classified into the shoulder, back, and leg support exoskeletons (adapted from [9]).

The integration of exoskeletons (EXOs) in industry holds promise, revolutionizing the way we work by enhancing the capabilities of the human workforce. Various types of such wearable assistive devices have been developed and are commonly categorized based on their application area as military, rehabilitation, or industry. Industrial variants of these systems are typically classified based on the method of their actuation, as activity (powered), or passive (mechanical, or pseudo-mechanical) systems. Passive industrial EXOs (i-EXOs), as shown in Figure 1, are more likely to be adopted by the workforce as these devices are compact, lightweight, and are easy to use. Each of these systems is designed to augment specific body regions (Shoulder/Back/Leg) or aid during specific tasks (like overhead assembly, lifting/bending, squatting) [1,2]. Use of i-EXOs offer several benefits, such as reducing the risk of musculoskeletal injuries, enhancing worker performance and endurance, and improving overall work quality, as displayed by detailed laboratory and field evaluations [3,4,5,6]. Despite these advantages, the adoption and implementation of exoskeletons in industry has been relatively slow. This may be attributed to various factors, including the need for customization to individual users and tasks, concerns regarding cost-effectiveness, and limited awareness and understanding of the devices among employers and workers. Furthermore, regulatory frameworks and standards for exoskeletons in the workplace are still evolving, posing challenges to their widespread implementation [7,8]. In addition to laboratory and field-based studies that examine the impacts of exoskeletons, it is imperative to prioritize the transfer of knowledge regarding their proper use to end-users. This approach can be crucial in ensuring that results from controlled evaluations are effectively translated towards real-world applications.

**Figure 2:**
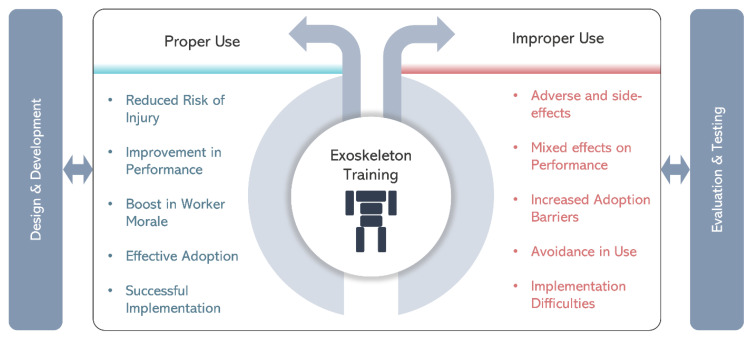
Illustration depicting the exoskeleton training being at the core of integration of the devices in industrial environments and its effects categorized according to proper/improper use of the devices.

Training plays a key role in integrating i-EXOs in industrial environments. If proper training is not provided to exoskeleton users, they may apply the devices in use-cases wherein they are not beneficial or may use them in improper manner (e.g., with a lack of fit by not adjusting device to anthropometric dimensions or using an incorrect assistance level). This may even lead to potential biomechanical and perceived effects that can impact their safety, performance, and overall well-being in the long run (Figure 2). Without adequate training, users may struggle with suboptimal posture and movement patterns while wearing the exoskeleton. Adaptation to EXO-assisted body movements requires effective motor learning. Specifically, improper alignment or excessive reliance on the exoskeleton’s assistance cause misalignment, increased joint loading, and adverse effects on wearers [10]. Users may also experience discomfort, fatigue, and reduced range of motion [11]. Additionally, without training, users may lack the necessary understanding of the exoskeleton’s capabilities and limitations, which can result in overestimation or underestimation of their own physical abilities, potentially leading to accidents or inefficiencies in task performance. From a perceived standpoint, the absence of training can result in frustration, anxiety, and decreased user confidence in operating the exoskeleton, inhibiting their willingness to fully utilize potential benefits of i-EXOs [12]. Proper training is therefore vital to ensure biomechanical alignment, reduce the risk of injuries, and enhance the user’s perceived comfort, confidence, and overall acceptance of exoskeleton technology.

The field of instructional design involves a systematic approach of developing effective and engaging training programs for product users [13,14,15]. Several methods are commonly employed in this process. Firstly, a thorough analysis of the target audience and their specific needs is conducted to identify learning objectives and create a learner profile [14]. This information forms the foundation for designing the training content and determining the most suitable delivery methods. Next, instructional designers often employ a variety of techniques such as storyboarding, concept mapping, and task analysis to organize the training materials and sequence the content logically [14,15]. Additionally, the use of formative and summative assessments can help in evaluating effectiveness of the training [13]. Throughout the process, collaboration with subject matter experts, pilot testing, and iterative design approaches can contribute to the development of a comprehensive and impactful training program for the device wearers [14].

Despite considerable research on evaluating effects of exoskeletons in the past 5 years, with numerous research [16,17,18,19,20,21] and review articles being published [1,2,3,10,22,23,24], there remains a noticeable lack of emphasis on the topic of training in scientific literature. This gap highlights a need to study and understand the importance of proper training for exoskeleton users. Given the intricacies involved in operating, adjusting, and maintaining exoskeletons, an instruction manual, although thoroughly prepared, may not suffice. Even after providing demonstrations, replicating movement patterns of experts may be difficult for novices. Comprehensive and in-depth training is essential to ensure user competence, minimize the risk of injury, optimize performance, and harness the potential benefits of these devices. There is a need to understand training protocols and develop best practices/guidelines to facilitate the safe and efficient integration of exoskeletons in occupational settings. The purpose of this review article was to understand currently used methods for training/familiarizing wearers of industrial EXOs. Objectives also included identifying key considerations for designing a comprehensive training program specifically for industrial EXOs. In addition, we also reviewed the effects of training and familiarization protocols on motor learning. Subsequent sections also list potential application of data-driven approaches and ergonomic evaluations in providing effective training to the workforce. Finally, a generalized training framework for users of industrial EXOs is provided along with potential applications of emerging technologies to augment training.

## 2. Review Methods

This narrative review involved searching relevant scientific literature on training/familiarization as well as motor learning and adaptations when using EXOs. While we mainly focused on studies related to design/development and evaluation of industrial EXOs where training was provided to study subjects, those with rehabilitation and mobility applications were also considered to understand effects of different familiarization protocols. Search methods involved conducting literature search in Web of Science, Google Scholar, and Scopus databases for journal articles, conference proceedings, and thesis published after 2010. The search was performed with the following keywords: “exoskeleton”, “evaluation”, “assessment”, “development”, “ergonomics”, “biomechanics”, and “wearable assistive device” in either/OR strategy. The number of published research/review articles specifically focused on “training” or having “training” within the title of such articles was very low, and thus we expanded our search to articles involving “training” and “exoskeleton” for rehabilitation purposes. We manually screened full text of each of the obtained articles and synthesized details relating to participant training (within the methods and procedures sections in most cases). After presenting a list of articles in the form of a table, articles were also subjectively rated by the authors based on the depth and comprehensiveness of provided training to study participants into categories of low/medium/high. Subsequent searches were also performed to review fundamental concepts in the field of instructional design and to identify emerging technologies that can be applied to improve EXO training programs. Findings of our review are presented and discussed in detail in the following sections.

## 3. Training and Familiarization Methods for Proper Use of Exoskeletons

Providing training in new technology for the workforce has been found to have significant positive effects compared to not providing training. Research has shown that training programs enhance employees’ knowledge, skills, and competencies, leading to improved job performance and productivity [25,26]. In addition, training plays a crucial role in promoting technological acceptance of technology among employees. Training helps individuals develop a better understanding of technology and its potential applications, increasing their confidence and reducing resistance to change [27]. Employees who receive adequate training are more likely to use the devices in the intended manner, enabling them to perceive the technology as useful. Moreover, training facilitates a smoother transition to the new technology, reducing errors, minimizing downtime, and improving overall operational efficiency [28]. On the other hand, a lack of training can lead to difficulties in adopting and effectively utilizing new technology, resulting in frustration, decreased job satisfaction, and increased resistance among employees. When employees do not receive adequate training, they may perceive the technology as complex, difficult to understand, and irrelevant to their job tasks, leading to lower levels of acceptance and utilization [29,30]. Besides perception, using wearable assistive devices requires adaptation to changes in body movement due to assistive/resistive forces. Subsequent sections focus on training related to operation and use of EXOs and provide an overview of approaches implemented to train study participants during exoskeleton evaluation studies, and effects of familiarization protocols on motor learning and biomechanical/perceived measures.

### 3.1. Training Novices during Exoskeleton Evaluation Studies

Evaluation-focused studies are conducted to assess the impact of i-EXOs on the human body and to estimate performance improvement/risk reduction. Such evaluations include recruitment of human subjects and simulation of industrial tasks. We reviewed articles involving design validation, laboratory, and field assessment of i-EXOs to understand currently used training methods. Findings from the review have been listed in Table 1 categorized based on the type of study (Validation/Lab/Field) and methods used for training (Duration and Tasks). Each of these studies were then categorized based on the identified level of depth, and comprehensiveness of the training session was categorized into three levels as High/Medium/Low level.

We reviewed studies that considered training sessions within their experimental protocol. Within the pool of articles, only one study conducted a laboratory evaluation to compare different familiarization methods, specifically reading instruction manual vs. observing a demonstration [31]. Participants of exoskeleton evaluation studies underwent a comprehensive training program on i-EXOs prior to the experiment [18,32], where participants were trained. In some cases, a separate session was dedicated for training participants on the use of the i-EXO [18,33,34], while in other studies, training was provided for a duration (either fixed, or until participants were comfortable/confident on using the device) prior to the experimental session. Among the reviewed articles, the studies wherein duration was fixed considered a time in the range of 2 min to 2 h [32,34,35,36]. In one field evaluation, participants were asked to wear and practice using the device for several days [37]. Upon coming for evaluation, participants were first educated with detailed instructions on safe use of the device [38] and a demonstration of its working [34]. In one study, an instructional video was shown to participants on proper way of donning/doffing the device [39]. The training tasks provided to participants often started with donning the i-EXO and adjusting the device to ensure proper fit. Participants were instructed to mostly practice the experimental tasks. Once proper fit was confirmed by the investigator, participants were asked to walk around until they were comfortable/familiar with the device [35,40]. This was followed by practicing the pre-determined task in each experimental condition [18]. During training, participants were also provided with the experience of max/min levels of assistance and were asked to select their preferred support level. Meanwhile, in other cases [40,41] the training included instructions on donning and doffing the exoskeleton, adjusting the assistance level, and maintaining proper body mechanics during tasks. Although not explicitly stated, the reviewed studies collectively show that incorporating training programs before experimental evaluations of i-EXOs can be beneficial for investigators as study participants need to acquire necessary motor adaptations to effectively perform intended body movements while being assisted with the devices. On the other hand, if sufficient training is not provided, study participants may perform body movements in a manner that the assistance from the device is not used to reduce the wearer’s effort during the task, or in worse cases, wearers may struggle or fight against the assistive torque, leading to increased demands.

A study that compared the effects of training (demonstration vs. reading an instruction manual) demonstrated that providing study participants with a live demonstration increased performance (and reduced errors) and users acceptance, as well as led to lower global and local perceived effort [31]. In fact, easiness of learning almost doubled when study participants were provided a tutorial vs. reading a brochure. While further evaluations are needed to assess the impact of training on the safety and performance, some can be expected based on current understanding of the design of exoskeletons [10]. Designing effective exoskeleton training for industrial workers requires consideration of various variables that can influence their performance and well-being. These include mainly the efficient delivery, depth of topics covered, comprehensiveness, and duration/timing, as well as the type of task. It is expected that a longer training duration generally leads to better biomechanical outcomes, as users have more time to adapt to the exoskeleton and optimize their movement patterns. The specific nature of the task is also important, as different tasks may require varying levels of physical exertion and repetitive movements [10,42]. A lack of proper exoskeleton training may lead to detrimental effects. Without adequate training, users may not fully understand how to operate and utilize the exoskeletons correctly. A recent study that implemented that unified theory of acceptance and use of technology (UTAUT) model to EXOs and showed explanations for 75% of variation in intention to use the devices [43]. Specifically, the findings denoted that effort expectancy (how easy it seems to use an exoskeleton) played an important role in model prediction.

**Table 1:** List of evaluation-based studies of industrial exoskeletons (i-EXO) that trained novice study participants prior to conducting experimental evaluations of the devices. Each of the listed studies has been rated according to the level of training provided to the study participants (L: Low, M: Medium, H: High).

Article Type	Exoskeleton	Sample Size	Training Duration	Training Tasks	Training Level (L/M/H)
Lab [18]	Fawcett Exsovest with ZeroG, EksoWorks Vest, FORTIS (Mechanical Arm, Back, Full-Body	12 (6♂, 6♀)	2 h session (5 min each condition), 2 min practice for each condition in actual session	Proper fit, Practice on each experimental condition, Selecting preferred assistance level.	H
Lab [44]	VT Lowe (Back)	12♂	~30 min	15–25 practice lifts with boxes of 0–25% body weight.	H
Lab [32]	VT Lowe (Back)	15 (13♂, 2♀)	~30 min	15–25 practice lifts with boxes of 0–25% body weight.	H
Lab [40]	Laevo (Back)	18♂	-	Proper fit, movement with walking and squatting.	L
Lab [41]	SPEXOR (Back)	26♂	~2 min	Movement to get habitual with device.	L
Lab [45]	Laevo (Back)	36♂	-	Proper fit, familiarization with performing tasks.	L
Lab [46]	Exo4Work (Back)	16♂	2 h	Two familiarization trials, task practiced seven times in each trial.	H
Lab [47]	StrongArm V22 (Back)	6 (4♂, 2♀)	15 min	Wearing and getting familiar with task.	L
Field [37]	Levitate Airframe (Shoulder)	6	Several days	Proper fit, familiarization with the work.	H
Lab [48]	Laevo (Back)	13♂	-	Wearing and getting familiar with task.	M
Lab [49]	SuitX BackX (Back)	10	-	Wearing and getting familiar with task.	M
Lab [34]	HeroWear (Back)	20 (15♂, 5♀)	1.5 h session	Demonstration, donning, unstructured movement with different levels of assistance.	H
Lab [38]	Noonee chairless chair (Leg)	17 (-)	-	Detailed instruction in the use (safe use) of the exoskeleton was practiced.	L
Lab [50]	Prototype (Shoulder)	8 (4♂, 4♀)	-	Practice the task and demonstrate the understanding.	M
Lab [51]	StrongArm Flx Ergoskeleton (Back)	20 (3♂, 17♀)	-	Instructions on performing the tasks.	M
Lab [52]	BackX (Back)	8	-	Wearing the exoskeleton few days before data collection.	L
Lab [5]	EksoVest prototype	12 (6♂, 6♀)	40 min	Proper fit, practice tasks, and encouraged to determine preferred postures.	H
Lab [53]	CEX (Leg)	20♂	-	Informed about wearing the exoskeleton.	L
Lab [35]	SPEXOR (Back)	14 (7♂, 7♀)	5 min	Proper fit, movement to get familiar with the exoskeleton.	L
Lab [54]	Active Pelvis Orthosis	5♂	~20 min	Familiarization with exoskeleton, tuning of assistance level based on comfort levels.	M
Lab [33]	Noonee chairless chair (Leg)	46♂	1st visit to lab (one session—30 min)	Getting familiar with wearing and handling the device, practice the tasks with/without exoskeleton.	M
Lab [55]	BackX Model AC (Back)	18 (9♂, 9♀)	3 h	Proper fit, familiarization with each condition, finding preferred support and postures.	H
Lab [56]	Laevo (Back)	9♂	10 min	Donning, movement, and familiarization with exoskeleton followed by performing tasks.	M
Lab [57]	Laevo (Back)	10	-	Introduction to exoskeleton, donning/doffing device until confident to perform task.	M
Field [20]	Noonee chairless chair (Leg)	4 (3♂, 1♀)	30 min (day before experiment)	Training with safety instructions on use of exoskeleton.	M
Lab [58]	Paexo Back (Back)	10 (5♂, 5♀)	20 min	Instructions, adaptation, and training on functions of exoskeleton and practice trial (for 5 min).	H
Lab [39]	BackX (Back)	30 (20♂, 10♀)	-	Watching instructional videos for the proper procedures to don and wear exoskeleton.	L
Validation [59]	E-Leg (Leg)	10♂	15 min warm-up phase before experiment	Practice (wearing, squatting).	L
Validation [60]	Custom prototype (Leg)	-	-	Orientation session, walking and squatting while holding a support rail. Fit was adjusted simultaneously.	M
Validation [61]	Custom Prototype (Leg)	10♂	Before experiment (unspecified)	Informed of the detailed test procedure and exoskeleton settings.	L
Lab [31]	SkelEx	36 (18♂, 18♀)	5 min for reading/demo, 3 min for testing participants on ability to install/adjust	Instruction manual vs. demonstration.	H

### 3.2. Familiarization Protocols and Their Effects on Motor Learning

A subsequent review was conducted to study familiarization protocols and metrics used to quantify motor learning across training sessions. The outcomes of the review can be viewed in Table 2. To summarize, the effects of familiarization protocols on benefits provided by EXOs were found to be mixed and depended on the design, purpose, task, and users. For instance, one study found that novices could not achieve motor strategies of experts even after three sessions [62]. A recent study incorporated a detailed familiarization protocol that consisted of loaded marches, don/doff, adjustment/assembly, warm- up, and muscle-activation exercises [63]. These tasks were carried out in three phases over a duration of ~3 weeks with the assistance of an evaluator. Interestingly, the findings showed that familiarization processes did not provide benefits of reducing metabolic cost and muscular benefits. However, an earlier study by the same group [64] showed that familiarization affected differently for different designs of the same EXO, and the adjustable design affected motor learning. The duration of required training for an ankle EXO was found to be more than the duration used for training in exoskeleton studies, and training type had a significant effect [65]. Another study conducted an assessment to determine the duration and number of training sessions required to get familiar with a soft back-assist EXO by evaluating changes in biomechanical parameters (such as postural stability and muscle activity) and recommended need for at least four sessions of 1 h duration [66].

As opposed to i-EXOs, those in the healthcare sector are often used for re-training lost body functions in patients, where therapists guide and assist. A prior study conducted a qualitative assessment by comparing two groups of therapists, where one was formally trained and the other was exposed to clinical practice [12]. Findings from the study highlighted a steep learning curve and perceived difficulties in implementation of the device. Benefits of using a familiarization protocol were found in a study evaluating perception and usability of an EXO for walking in study participants that were provided training [67]. A detailed step-by-step method consisting of 11 steps for training patients on proper use of the devices has been proposed in a prior study [68], and similar protocols could be developed for training users of their industrial variants. Studies also demonstrate the use of biomechanical metrics as indicators of familiarization. For example, gait parameters of stride duration, mediolateral deviation, and polygon of support area were found to be good familiarization indicators for gait-training EXOs. Another study developed a method to replicate body movements of therapists in patients and promote correct motor learning [69].

**Table 2:** List of studies that implemented familiarization protocols to study their effects on motor learning when performing tasks while assisted with an exoskeleton (EXO).

Article	EXO (Primary Purpose)	Study Goals	Sample Size	Familiarization Protocol	Outcomes
[64]	UPRISE Gen 3.0, 4.0 (Military)	To assess differences between customizable (Gen 3.0) and adjustable (Gen 4.0) designs of EXO.	Dataset 1: 3♂ soldiers, Dataset 2: 3 healthy participants	Gen 3.0: 3 h daily sessions for 9 days distributed over 2 weeks. Gen 4.0: 14 days of training (around 1 h 30 min) distributed over a period of 4 weeks.	After familiarization, users reduced their MCW when using the customized but not adjustable EXO. Adjustment of the EXO affected user and motor learning.
[63]	UPRISE Gen 4.0 (Military)	To understand changes in metabolic cost of walking after a familiarization process.	13 (12♂, 1♀)	~14 days of tasks (~1–1.5 h) with an evaluator. Distributed practice and gradual progression of loads and difficulty across three phases. Tasks included loaded marches, don/doff, adjustment/assembly, warm-up, and muscle-activation exercises.	Familiarization process did not provide benefits of reducing the metabolic cost of walking and muscle activation.
[62]	Guardian XO 2019 prototype (Industrial)	Adaptation of novices to EXOs and comparison with experts.	11♂ (6 novices, 5 experts)	Novices: initial familiarization and three subsequent gait sessions. Experts: one gait session.	Novices demonstrated progress but could not achieve similar motor strategies as experts even after three sessions.
[68]	ExoAtlet (Rehabilitation)	Propose steps for training patients on using the EXO.	1♂	Validation: Electroencephalography (EEG) topographic maps, pressure insoles, and discomfort.	Training was beneficial in clinical settings.
[70]	TWIN (Rehabilitation)	To provide four biomechanical metrics as familiarization indicators.	5	Five walking bouts. Evaluation measures: gait parameters, support area, and muscle activity.	Stride duration, mediolateral deviation from a straight path, and polygon of support area were found to be good familiarization indicators.
[71]	Soft hip EXO (Military)	To study metabolic adaptations over training sessions.	8♂ military cadets	Five training sessions within 20 days. Task consisted of loaded walking (with ~20 kg).	Percentage benefits of EXO in net metabolic cost improved across sessions from −6.2 ± 3.9% (session one) to −10.3 ± 4.7% (session five).
[72]	Prototype (Rehabilitation)	To conduct wearability evaluation of EXO.	15 healthy individuals (7♂, 8♀), 2♂ stroke survivors	Trials with donning/doffing the device. Evaluation included donning/doffing time and usability.	Participants were able to independently don and doff the device after four practice trials.
[31]	SkelEx (Industrial)	To assess effects of media used for familiarization.	36 (18♂, 18♀)	Instruction manual vs. demonstration. 5 min for reading/demo, 3 min for testing participants on ability to install/adjust.	Live tutorial led to higher task performance and user acceptance and reduced global and local perceived effort as well as errors.
[67]	TWIN EXO (Rehabilitation)	To study effects of familiarization protocol.	9 (6♂, 4♀)	Proposed protocol included: preparation, tutorial, exoskeleton session, and ending. assessment that included System Usability Scale, NASA Raw Task Load Index, and surveys.	The protocol was found to be beneficial for improving familiarity.
[66]	CORFOR soft back EXO (Industrial)	To characterize the familiarization procedure and determine the time for stabilizing biomechanical parameters.	18♂	Six familiarization sessions of 1 h. Measurements included kinematics, posture stability, perception, muscle activity, and performance during lifting tasks (8 kg box).	Recommend four sessions of 1 h to stabilize parameters.
[65]	Bi-lateral ankle EXO (Mobility)	To understand the effects of training and type of training on metabolic cost.	15 (10♂, 5♀)	Three training groups with variation of Low, Medium, and High in device behavior.	Training required more exposure than typical studies of 109 min of assisted walking. Low variation group needed 2× duration of moderate group. High group never reached expertise. Metabolic cost reduced by 39%, and training contributed to half of the benefits.

## 4. Limitations of Current Training Methods and Implementation Challenges

Review findings indicate a wide variation in the training procedures (duration and tasks) for training study participants during EXO evaluation studies. For instance, duration ranged from as low as a few mins per experimental task up to several days depending on the complexity of the experiment, aims of the experiment, the design of the EXO being evaluated, and the application area. While in some cases participants were trained in donning/doffing, proper fit, and safety procedures, others mostly focused on specific experimental tasks (Table 1). As observed from Table 2, effects of the type, duration, and comprehensiveness of the familiarization protocols were observed on physiological parameters during biomechanical assessments. Differences in training protocols may have affected the level of adaptation across study participants, as well as between studies. This implies that EXO evaluation studies require standardized training protocols so that the outcomes are more generalizable across studies. It should also be noted that reviewed studies (Table 1) involved novice participants that were recruited from the university population, and there could be several challenges when training industrial workers. For example, depending on the education level of workers, learning about using the device may first require providing fundamental knowledge about the function of the devices. Similarly, demonstrations and familiarization protocols may require instructor interventions as well as assessments to test whether these devices are being properly used.

Designing proper training programs for users of i-EXOs poses several challenges. One significant challenge is the customization of training to accommodate individual differences in anthropometry, physical abilities, and prior experience with exoskeletons [23]. These factors must be thoroughly assessed to tailor the training program effectively. Another challenge is achieving optimal fit and alignment of the exoskeleton to the user’s body. Exoskeletons come in various designs, and anatomical variability among users can make it challenging to ensure a proper fit. Although alteration in design may be controllable, especially during implementation phases, ensuring proper fit prior to use may help in reducing such side effects. Besides relying on subjective measures (perspectives from users) about the fit, objective methods could be utilized. To elaborate, along with simpler verification based on body size (anthropometric dimensions) and provided instruction manuals (that often provide adjustability steps based on body/segment lengths), body shape considerations are also needed. We recommend that instructors in exoskeleton training focus on structural components of the device, their adjustment, and their relation to discomfort caused by high-pressure spots at interfaces/attachments. After a low-fidelity testing (subjective rating of comfort), a detailed assessment may be conducted using objective tools such as pressure mat systems to assess the presence of such discomfort regions [73,74,75]. Similar studies have also provided insights on user comfort level based on body demands and range of motion [76,77]. Inputs from such studies (such as setting an optimal assistance level to minimize discomfort and side effects) could play a crucial role in the development of instructional materials for specific tasks/conditions in which the i-EXO in consideration may be helpful. The following section provides a more detailed account on the role of ergonomic evaluations in informing design of training programs.

## 5. Role of Ergonomic Evaluations in Exoskeleton Training Design

The field of ergonomics involves the study of human interactions with products, processes, and the environment to optimize system safety and performance. Often, ergonomic evaluations include obtaining qualitative (like comfort and usability) and quantitative (such as kinematics, kinetics, muscular demands, and metabolic rate) measures to estimate the impacts of interventions (i.e., EXO) on the human body. Earlier controlled assessments have provided a detailed account of tasks where EXOs can be beneficial and those where EXOs are known to increase demands (side- and adverse-effects). A recent review [10] has stated that the majority of side effects (discomfort and reduced usability being the most prominent) are related to incorrect fit, uncomfortable materials (e.g., straps, belts, plates), or misalignment between i-EXO and human joints. Such conditions may cause variation in distribution of assistive forces/moments, slippage of attachments during work, or increased joint loading in other body regions. Proper donning/doffing and use of EXOs can be evaluated by testing motor learning patterns of novice users and comparing biomechanical measures to those of experts. Training can incorporate biomechanical assessments to study forces, movements, and loads on the human body (for instance, calculating low-back compression force at the L5-S1 joint). This can help in obtaining insights into optimal movement patterns (comfortable range of motion of upper limbs) and muscle-activation strategies (increase/decrease in muscular demands within specific muscle groups). Previous studies that evaluated temporal effects of using i-EXOs denote that the devices may be helpful in reducing fatigue [6,34,78], yet longitudinal and field studies have shown mixed outcomes [3].

Few efforts were found on understanding the effects of adaptation/learning during early phases of using EXOs as depicted in Table 2. One study [31] involved a lab-based study that compared the effects of familiarization protocols of providing a live demonstration vs. reading an instruction manual. As expected, the findings showed that providing a demonstration was beneficial in improving performance as well as usability and reducing physical demand. In another study, differences in walking patterns between six novices vs. five expert users were compared after they wore a whole-body active EXO in a recent article [79]. The outcomes showed significant differences in spatial (25% less step/stride lengths, and ~8° less knee/hip flexion among novices) parameters during walking. These differences decreased after three training sessions showing the benefits of training EXO users. Meanwhile, another study showed that during early adaptation phases, users may require additional cognitive resources during movement while wearing an EXO, as interpreted from increased visual reaction time [80]. A more detailed study was also conducted to study adaption of naïve users to an ankle-assist EXO by offering different levels of variation in training (Low, Medium, High) [65]. With moderate training, large benefits were displayed for customized assistance mode as shown by ~39% reduction in metabolic rates compared to walking with the EXO turned off. Furthermore, the study also showed that types of training can significantly affect training duration. In another study about evaluating motor adaptation of an ankle EXO controlled using muscle activity, recruitment of soleus muscle decreased by ~35% after practice [81]. Moreover, study participants retained adapted locomotor pattern, as assessed by similar muscle activity, kinematic, and kinetic forms between different sessions. These studies not only highlight the benefits of providing training to EXO users, but also show the role of ergonomic/biomechanical evaluations in developing training programs.

## 6. Comprehensive Framework for Training Exoskeleton Users

**Figure 3:**
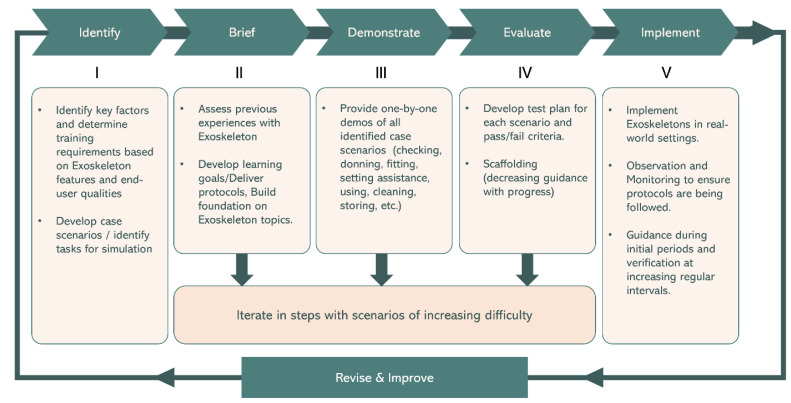
Illustration depicting the IBDEI (Identify, Brief, Demonstrate, Evaluate, Implement) exoskeleton training framework for guiding the workforce towards effective and safe use of industrial exoskeletons (i-EXOs).

The successful integration of i-EXOs into the workplace requires a comprehensive and effective training program. For instance, a prior study mentioned a familiarization protocol consisting of the following steps: Demystification, Technics, Potential, Limits, Donning/Adjusting/doffing, and Free experience (without industrial constraints) [31]. The study provided a 7-level familiarization chart, and suggested that subjects need to reach a level of four prior to using i-EXOs [31]. Fundamental theories and models in the field of instructional design were reviewed to understand methodologies used to develop training programs. According to the principles of effective instruction, learning is promoted when (a) it is problem-centered, (b) prior knowledge is activated, (c) new knowledge is demonstrated, (d) applied, and (e) integrated in the real world [13]. These principles can be related to designing training protocols for specific applications. Based on the instructional design practices, we propose a training framework abbreviated as IBDEI (Identify, Brief, Demonstrate, Evaluate, Implement) for training the workforce on i-EXOs [13,25,29,82]. This five-step approach (Figure 3) encompasses the identification of user needs, delivery of fundamental knowledge, hands-on demonstrations, evaluation of performance, and real-world implementation.

### 6.1. Identifying Key Factors and Training Requirements

Planning a training initially involves understanding the target user group and their specific needs [15]. In order to identify important factors in designing an exoskeleton-specific training, we reviewed studies on exoskeletons focusing on the design of the devices, their usability, wearability determinants, and adoption barriers [12,72,83,84,85]. i-EXOs are designed to be worn by the working population. Literature demonstrates the application of these devices in industrial tasks such as assembly [17], construction [57], and healthcare [56] where repetitive and sustaining postures (such as flexed trunk during bending or holding hands above head during overhead work) are common. Furthermore, end-user specific factors such as physical abilities, job tasks, and prior experience with exoskeletons should be assessed prior to developing a training. Although the end-users of i-EXOs, being a manual workforce, could be assumed to represent specific characteristics (such as education level), such characteristics may vary depending upon the industry sector. For example, user requirements in manufacturing (male dominant workforce, majority of overhead and/or lifting tasks, rugged environments) vs. healthcare (female-dominant workforce, high-paced work, sterile environments) may be fundamentally different (including the use of i-EXOs). Thus, the comprehensive instruction on the correct operation, adjustment, and maintenance of the i-EXO can vary based on the use case scenario of end-users.

**Figure 4:**
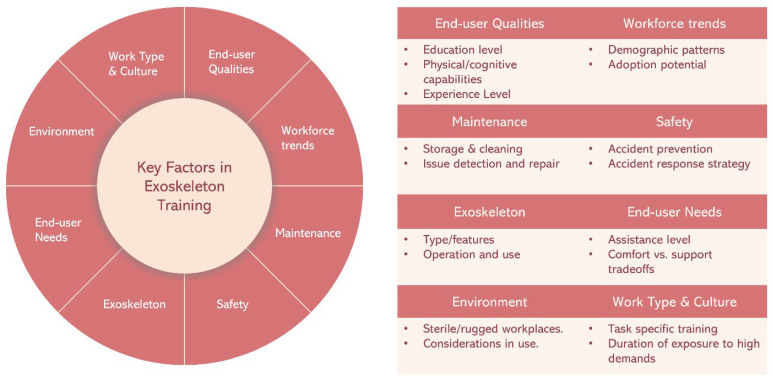
A schematic showing an overview of key factors to be considered in developing proper training for exoskeleton users.

Improper fit and lack of adjustment could cause detrimental effects on the wearer’s body [86]. Thus, training should cover proper fitting procedures, adjustment of device joint stiffness and assistance levels, troubleshooting common issues, and emphasize education on ergonomics and body mechanics to promote proper movement patterns, which depend highly upon the users and type of task during their routine work, as well as the work culture (level of high demands and exposure time). Although i-EXOs aim to reduce the risk of musculoskeletal injuries, if incorrectly worn or used, the assistance provided may impede natural body motion or harm the otherwise healthy joints of users [10,11,87]. Similarly, safety protocols and emergency procedures should also be emphasized, especially during rare cases if the structure of the i-EXO breaks or the mechanism malfunctions, leading to unintended forces on body joints and body movement [8]. Considerations are also required for covering instances and action plans for accidents occurring during use of i-EXOs. Due to a wide variation in the type and design of i-EXOs, developing a regime that fits every case may be infeasible; hence, developing a systematic approach to training design is needed. In summary, these eight key factors as depicted in Figure 4, namely, end-user qualities, workforce trends, maintenance, safety, work culture, end-user needs, environment, and work type could be crucial in developing training for exoskeleton users.

To understand the individual user needs and expectations, tools such as questionnaires and one-to-one semi-structured interviews may be used [13,26,29]. Individual problem statements can then be designed to target each of the challenging aspects required in effectively and safely performing the tasks using i-EXOs. Problem statements can be generated based on some of the identified key factors, and Table 3 depicts a list of such potential statements that may be used for developing case scenarios to train i-EXO users.

### 6.2. Briefing, Demonstration and Evaluation

The second step, “Brief” step focuses on delivering fundamental knowledge and educating users on the basics of exoskeleton operation, adjustment, and maintenance. This may include providing information on the correct fitting procedures, adjustment of joint stiffness and assistance levels, as well as troubleshooting common issues of i-EXOs. Particularly, fundamental operating principles of the device can be delivered during this phase (such as checking mechanism/actuator functionality and assistance levels prior to donning). Training materials and instructional strategies may also include mixed media (video/audio descriptions of taught content) and must be pre-designed to effectively deliver the essential knowledge to users [26].

In the demonstration step, learners should be provided with one-by-one demonstrations of task scenarios that are based on the identified key factors (Figure 3), such as fitting the exoskeleton to the user’s body, setting appropriate assistance levels, and executing the task safely and efficiently. Such demos can be conducted by a trained instructor or by individuals with previous experience in using i-EXOs. Demos should guide learners through the entire procedure of donning, setting assistance, using, and doffing. Furthermore, simulations of activities that may not be evaluated can be demonstrated under controlled circumstances (such as response strategy in the event of mechanism malfunction during use).

Evaluation is a crucial step in the training framework, where a test plan is developed to assess the user’s performance and understanding of the training content [88,89] as per the discussed considerations for the role of ergonomic/biomechanical evaluations in ensuring training effectiveness. This evaluation process may include hands-on assessments, practical exercises, and simulated tasks. Gamification alternatives can also be explored to improve motivation and learning experience [90]. Specifically, learners may be asked to perform the demonstrated activities under vigilance from an expert/instructor with appropriate interventions to correct learners’ performance. The training can be designed with scaffolding, which involves gradually reducing guidance as users progress through increasing difficulty levels. Due to wide variations in commercial EXO products (active/passive, shoulder/back/leg, rigid/soft) as well as industrial activities, assessments can be designed on a case-by-case basis depending on the type of i-EXO and specific task under consideration.

The three phases of briefing, demonstrating, and evaluation, treated as a module, can be iterated for the number of identified problem statements (e.g., optimizing assistance for individuals, visual inspection of mechanism, etc.). Each such module can be defined based on increasing level of difficulty (with the easiest ones completed first). After completion of each module, appropriate tests must be provided to ensure that learners can independently solve each of the defined problem statements. More complex tasks may be assessed at this stage with the help of biomechanical data-acquisition methods (such as measuring metabolic rate differences or muscle activation [6,78,91,92,93]) to ensure that the devices are beneficial. Lastly, feedback from assessments and user observations helps identify areas for improvement in subsequent training modules.

### 6.3. Implementation in the Real World

The final step in the framework focuses on implementing the trained skills in real-world settings. Users may be observed/provided with continued guidance to ensure proper adherence to protocols and safe operation of the exoskeleton. Observations and user feedback at this stage may also be used to further enhance and refine the training program for future iterations [29]. With the proposed framework, training programs for i-EXOs can be designed to address specific factors and challenges, ultimately enhancing user competency and facilitating their safe and effective integration into the workplace.

## 7. Data-Driven and Emerging Technologies for Augmenting Exoskeleton Training

Performing tasks safely and effectively with an i-EXO depends on the ability of the individual to learn the needed changes in motor skills when wearing the device. Emerging technologies such as Virtual Reality (VR) or Augmented Reality (AR) can be utilized for simulating realistic tasks [94]. For example, VR simulators have been found to be effective in improving training on surgical robotic systems [95]. Prior efforts demonstrate the use of a Virtual Reality (VR)-based framework to test, experience, and train users based on a set of feedback modalities [96]. The simulation platform enabled users to select parameters for the simulation, which provided feedback in the form of visual, vibro-tactile, and force-feedback to the users. In addition, outcomes on motion and metabolic exposures were also provided to users, along with trajectories which may assist in reducing adaptation time while also providing real-time feedback. In another study, a haptic-based sensation transfer system was developed to migrate haptic and kinematic feeling of experts of i-EXOs to novices [97]. Similarly, a simulator-style device representing different types of i-EXOs, and their actuation capabilities, can be developed, which can be used to train the workforce in the real world. Such a simulator can incorporate features from a diverse set of i-EXOs in the market along with adjustable parameters that can enable training the workforce on different types of devices. Along with helping users to get trained on different types of devices, such a system can also be helpful in identifying devices in the market that are better suited to the user characteristics/preferences and the task.

Sensors and data-driven approaches can be utilized to estimate real-time physical demands by recording measures like muscle activity, trunk motion, and stability as the trainee performs the tasks, as demonstrated in previous evaluation-based studies [98]. More importantly, this can be utilized to identify and predict instances (postures or movements) and demands for selected tasks, device parameters, and user characteristics that can lead to detrimental effects on the wearer’s body. In addition, real-time fatigue-level prediction and forecasting models can be implemented to predict temporal effects, as described in our previous work [99]. Providing this feedback to the trainer can drive interventions, such as correcting body postures/movements or even the selection of appropriate device parameters. With recent technological developments in digitization, intelligent tutoring systems can be developed and implemented to provide personalized training to users [100]. For instance, data from trainees related to performance or physiological measures can be collected during training sessions and compared with previously established standards (e.g., with experts) to customize delivery and pace of the training sessions. A training platform utilizing integrated mechanisms, sensing, Artificial Intelligence (AI), and VR/AR was proposed in a recent study where performance evaluation was conducted throughout as workers performed tasks [101]. Another advantage of using VR/AR-based systems is that they can provide real-time visualization of training progress, performance, as well as provide guidance and instructions. To summarize, AI-assisted training can be beneficial by providing customized training based on individual experiences/skill levels and improving overall outcomes.

## 8. Study Limitations

Due to the rapid growth in exoskeleton research and development, the implementation requirements of the devices have not been thoroughly explored. Specifically, topics related to training of i-EXO users in actual workplaces have not yet been explored in academic literature. Although this article considered articles that considered training novice users of i-EXOs, many such studies included training/familiarization protocols to ensure motor adaptations prior to experimental assessments. As training itself was not an objective, many details regarding the training were observed to have been omitted. Only a few studies with primary aims of evaluating effects of familiarization protocols were found in the literature. Future research in the field of training for i-EXO users holds significant potential for enhancing user safety, performance, and overall experience. One important direction for future investigation is the development of personalized training approaches that consider individual user characteristics and needs, such as anthropometry, physical abilities, and prior experience. Additionally, further research is needed to explore the long-term effects of exoskeleton-provided training (vs. not providing detailed training) on user performance, musculoskeletal health, and work-related outcomes. Investigating the optimal timing and duration of training programs, as well as the potential benefits of ongoing training and refresher courses, can provide valuable insights for designing comprehensive and sustainable training protocols. Furthermore, the integration of emerging technologies (such as the use of motion capture systems), in training programs warrants exploration to enhance engagement, simulate realistic work scenarios, and facilitate skill acquisition. In this study, a framework has been proposed for training i-EXO users. Although the method is derived from foundational concepts in instructional design, the proposed framework needs to be tested by conducting detailed evaluations to ensure the retainability of the learnt topics. Future research efforts in these areas can contribute to the advancement of training practices and optimize the utilization of i-EXOs across diverse work settings.

## 9. Conclusions

Educating workers on the proper use of assistive devices plays a vital role in ensuring the safe and effective integration of i-EXOs into the workplace. Review findings indicated variations in training methods (duration and familiarization tasks) across evaluation-based studies. Training was provided for donning/doffing, ensuring proper fit, body movement, experimental tasks, setting assistance levels, and safe use. Means of instruction included instruction manuals, videos, and demonstrations by instructors. Studies that assessed the effects of familiarization protocols showed that novice users may require up to four sessions to achieve motor strategies of experts. On the other hand, evaluation-based studies often considered training sessions of much shorter durations, indicating the need to test motor adaptations of study participants prior to testing. Incorporating inputs from ergonomics and biomechanics can enhance the design of training and adaptation to using the device, ultimately ensuring proper movement patterns and safe operation. Considerations such as understanding the target user group, providing comprehensive instruction on operation and maintenance, emphasizing ergonomics and body mechanics, and utilizing a combination of instructional methods contribute to the success of training programs. Training sessions can be assisted with ergonomic/biomechanical evaluations (such as comparisons between novice/experts) to gain insights on motor learning patterns of novices and could be potentially helpful in determining the optimal duration and instructional depth across different types of EXOs and tasks. Future studies can integrate technologies such as simulators, VR/AR, and sensor-based data-driven approaches, including the use of machine learning and AI-assisted guidance for improving training outcomes.

## Figures and Tables

**Table 3:** List of potential problem statements to be used for developing training protocols.

No.	Problem Statements for Developing Problem-Centered Instruction Protocols
1.	How to determine and set required assistance levels for actuators of the exoskeleton?
2.	How to adjust the exoskeleton to varying body sizes/shapes?
3.	How to detect and test if the actuators are working properly?
4.	What is the step-by-step process of donning, and doffing the exoskeleton?
5.	How to engage/disengage actuator assistance of the exoskeleton?
6.	How to perform tasks by using assistance provided by the exoskeleton?
7.	How to determine whether the exoskeleton requires maintainence, or repairs?
8.	What is the procedure for cleaning the exoskeleton without damaging?
9.	What is the procedure to safety remove the exoskeleton in the event of a breakdown?
